# Use of Erbium Laser in the Treatment of a Patient with Acute Purulent Periostitis and a Resistant Form of Primary Immune Thrombocytopenia

**DOI:** 10.1155/2019/8260605

**Published:** 2019-08-19

**Authors:** Elena Larionova, Svetlana Tarasenko, Elena Morozova, Ekaterina Diachkova

**Affiliations:** Department of Dental Surgery of the Institute of Dentistry, Sechenov First Moscow State Medical University (Sechenov University), Russia

## Abstract

In our article, we described a clinical case of a patient with an acute periostitis of the maxilla with a comorbidity (primary immune thrombocytopenia, resistant form) who was treated using laser technology allowing us to provide good results: an intensive regeneration process and a decreased risk of developing infection complications and unpleasant signs for the patient, such as postoperative edema and intraoperative and postoperative pain syndrome.

## 1. Introduction

Normally, the platelet content is 150‐400 × 10^9^ per liter. A decrease in their number below 100 × 10^9^ per liter (thrombocytopenic syndrome) is a risk factor for the development of severe bleeding especially during surgical interventions when the integrity of the blood vessels is violated. Reducing the number of platelets below 30‐50 × 10^9^ per liter contributes to the appearance of spontaneous bleeding [1, 2].

The most common form of thrombocytopenia is the primary immune thrombocytopenia, which is an autoimmune disease characterized by the production of antiplatelet antibodies leading to a decrease in the number of platelets [1, 2]. During this process, a sharp shortening of the life span of the platelets from 7 to 10 days till a few hours occurs. Disease often (approximately 40% of cases) is the cause of hemorrhagic syndrome in clinical practice, significantly affecting the patient's quality of life and undermining personal self-esteem and social integration. The main reason for the bleeding in this situation is a quantitative deficiency of platelets and their functional inferiority. At the same time there is a disruption of the vascular link of hemostasis due to the disruption of the trophism of the endothelial lining of blood vessels [3].

The complexity of the situation in these patients is due to an opportunity to treat only postoperative complications but not their preventive procedures. There are different data in special literature about the methods of solving the problem of bleeding after dental manipulation: tranexamic acid, extract of green tea, and others [4, 5].

Schaffer et al. in 2016 have reported about the results of their study; they identified major barriers to performing oral care to people with bleeding disorders such as the economic side of treatment, which is highest in the list of reasons for the lack of access to dental care. Lack of skilled dentists and the anxiety of patients themselves were also a huge problem, although a significant number of doctors have expressed a desire to have the opportunity and skills to provide good oral health outcomes to their patients [6].

The aim of our research was the evaluation of the effectiveness of an erbium laser in the provision of surgical dental care to a patient with acute periostitis and primary immune thrombocytopenia on an outpatient basis.

## 2. Clinical Case

Patient K., 26 years old, was diagnosed the acute purulent periostitis of the upper jaw on the left side. Comorbidity was a primary immune thrombocytopenia, resistant form, which has appeared from the year 1991. The patient takes courses of glucocorticosteroid medicine, eltrombopag. He refuses splenectomy.

The patient has agreed to the use of material with his data in oral and written forms according to the European Medicines Agency Guidelines for Good Clinical Practice and the requirements of the local ethics committee.

From anamnesis, the patient complained of severe sharp pain in the upper jaw on the left side. About 4 days ago, the patient had noted pain, which increased when biting in the area of the 2.5 tooth, previously treated endodontically. He has applied to the city dental clinic and private dental clinics in his place of residence (Lipetsk). The patient was denied help four times due to comorbidities, and antibacterial and anti-inflammatory therapy was prescribed. The inflammatory process increased, and the pain intensified and became diffuse. The patient turned for help to hematologists of the special federal hospital, where the following premedication was performed alternately and intravenously: calcium gluconate 10%, 10 ml; dicinon, 4.0 ml; ascorbic acid 5%, 5 ml; and prednisolone, 60 mg.

**Figure 1:**
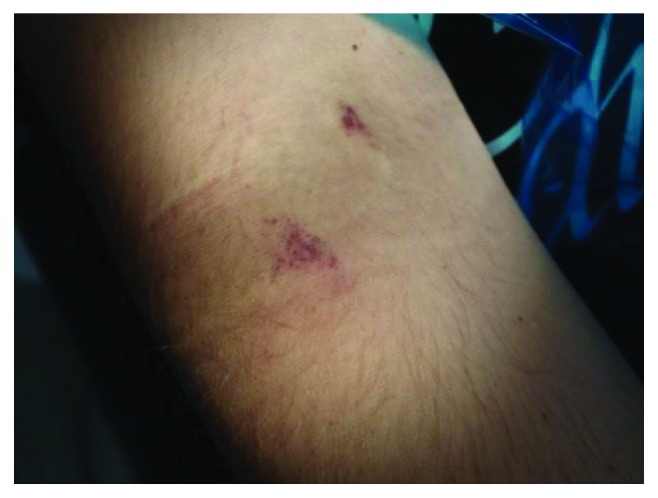
Petechiae on the skin in the area of the elbow bend.

**Figure 2:**
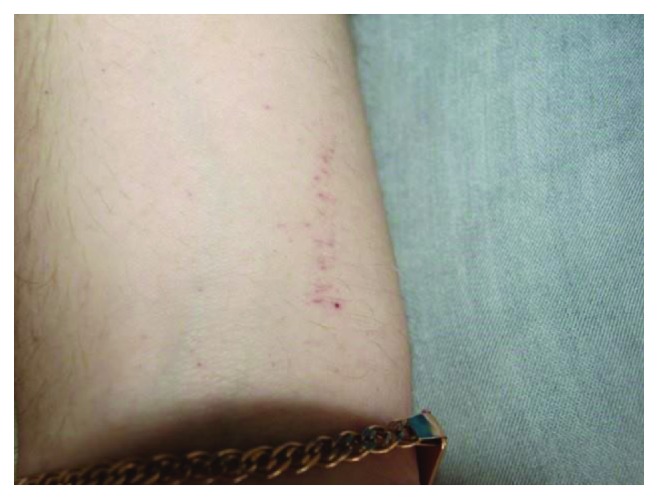
Petechiae on the skin of the forearm.

**Figure 3:**
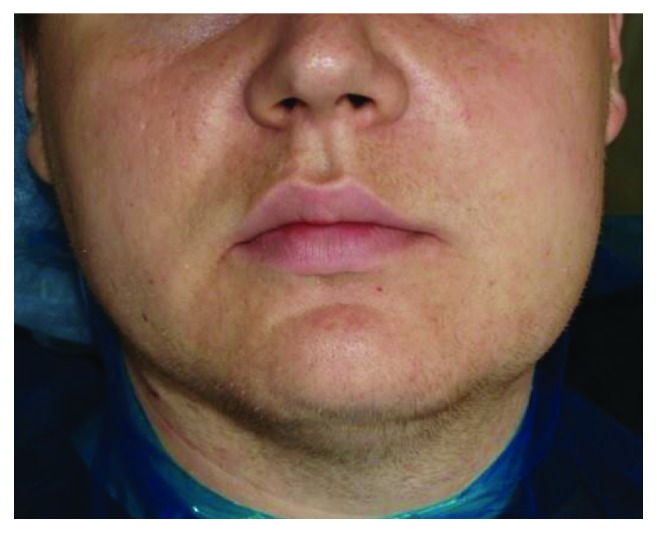
Collateral edema of the soft tissues of the left buccal region.

During examination on the skin of the surface of the elbow, shoulder, and forearm, multiple petechiae were determined (Figures 1 and 2). The collateral edema of the soft tissues of the left buccal region was visualized, and the lymph nodes of the left submandibular and submental regions were slightly painful on palpation, mobile, and enlarged to 1.5 cm in diameter (Figure 3).

In the oral cavity, the bulging of the transitional fold was determined in the 2.4, 2.5, and 2.6 teeth areas, the mucous membrane was hyperemic and edematous, palpation was sharply painful, and accordingly, the fluctuation focus was palpated near the 2.4 tooth. Tooth 2.5 was under a metal-ceramic crown, and percussion was sensitive.

In the preoperative period, the patient underwent clinical and X-ray examination, laboratory blood tests (general clinical blood test and determination of bleeding time by the Duke method and clotting time of whole blood according the Moravits method), and a consultation with a hematologist.

**Figure 4:**
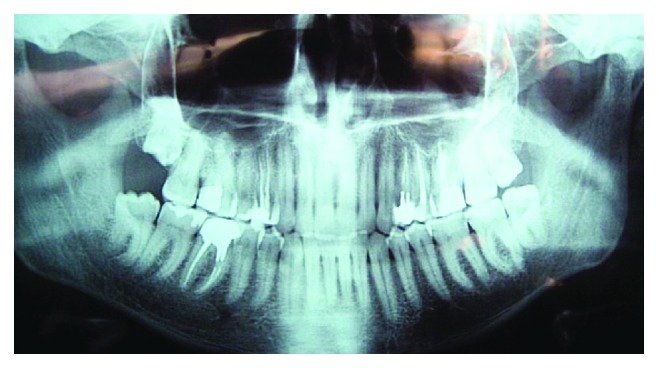
Orthopantomogram of patient K.

The results of laboratory blood tests were as follows:

1. Platelets were 2 × 10^9^/l (on treatment). Duration of bleeding based on the Duke method was 15 minutes and 30 seconds and clotting time was 4 minutes and 30 seconds.
2. On X-ray examination, bone thinning was found at the apex of the 2.4 tooth root with clear contours up to 0.6 cm in diameter; the root canal was sealed on 2/3 (Figure 4).

After a clinical and radiological examination, a decision was made for reendodontic treatment of the 2.4 tooth, cutting a subperiosteal abscess. The orthopedic structure was removed from the 2.4 tooth, and the therapeutic dentist performed a disclosure and temporary filling of the 2.4 tooth canal. A temporary plastic crown was made on the tooth.

In this case, we used an erbium laser with a wavelength of 2940 nm, which can be used on both soft and bone tissues.

**Figure 5:**
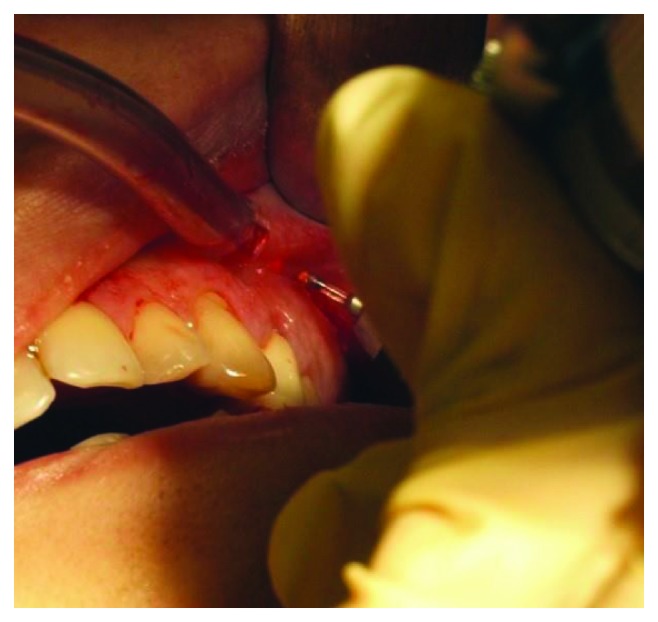
Cutting the subperiosteal abscess using an erbium laser.

**Figure 6:**
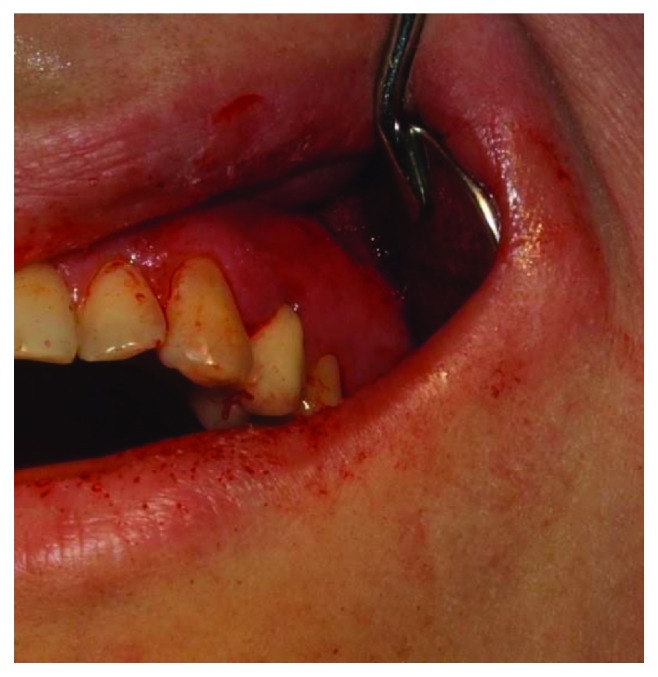
View of the wound immediately after the intervention.

Under infiltration anesthesia and using an erbium laser with an irradiation energy of 300 mJ and a frequency of 10 Hz without water-air cooling, an incision was made along the transition fold in the 2.4, 2.5, and 2.6 teeth areas, bluntly penetrating deep into the inflammatory focus, and a rich purulent discharge was obtained (Figure 5). The wound was treated with an erbium laser (Figure 6). Pronounced bleeding during surgery was not determined. Hemostasis time was 95 seconds. The patient was prescribed a broad-spectrum antibiotic therapy and antihistamine medicine. Postoperative bleeding was not observed. The patient noted minor pain in the postoperative area, not requiring the use of pain killers. Collateral edema of the soft tissues did not increase in the postoperative period, with a decrease in postoperative edema from 2 days after surgery.

**Figure 7:**
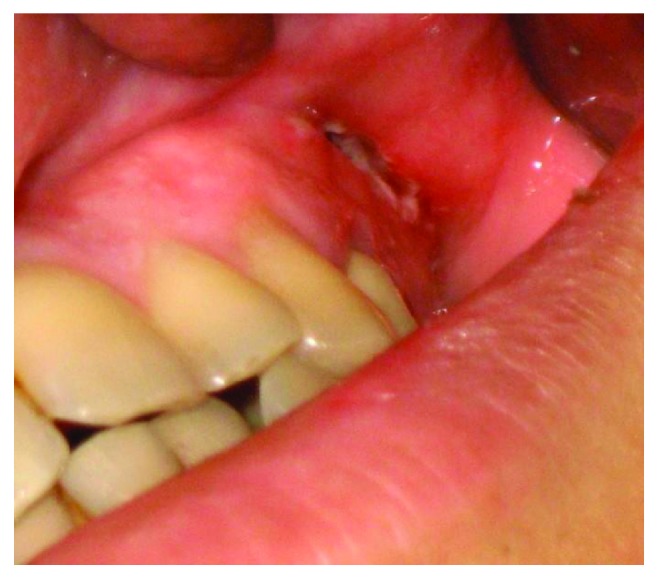
Wound on the 3rd day after drainage removal.

On the third day after the surgery, a slight collateral swelling of the soft tissues of the left buccal region was determined, and there was no drainage discharge. Drainage was removed, and further healing of the wound proceeded without features (Figure 7).

## 3. Discussion

When providing surgical dental care, the patients with primary immune thrombocytopenia have a risk of developing intra- and postoperative bleeding and also a decrease of the regeneration processes associated with taking glucocorticosteroids prescribed for treating the main disease and the deficiency of platelet factors [7]. Surgical dental care for patients with damaged hemostasis, as a rule, is performed in the hospital after a long medical preparation, which is difficult when the patient's complaint on the sharp pain is associated with an acute inflammatory process.

Until now, this cohort of patients is deprived of the possibility of obtaining timely and high-quality dental therapeutic and preventive care in outpatient conditions, which largely determines the high prevalence of inflammatory diseases of the maxillofacial area, their severe course.

Recently, laser technology has found more and more medical applications [8–10]. Dissecting the tissue, the laser beam simultaneously coagulates blood vessels on the margins of the wound, promoting hemostasis [11, 12].

We evaluate the possibility of using this laser technology. Cutting the tissue, the laser beam simultaneously coagulates the vessels on the wound walls, promoting hemostasis. In addition, postoperative edema is minimal and the intensity of the intraoperative and postoperative pain syndrome decreases [4, 10]. Laser radiation has a bactericidal effect on the pathological microflora in the operative zone, stimulates metabolism and tissue regeneration, and the increases the oxygen content in tissues, accelerating their early formation and preventing the formation of coarse postoperative scars [5, 10].

The use of a surgical laser in patients with impaired platelet hemostasis is designed to reduce the duration and volume of preoperative drug therapy and reduce the risk of secondary bleeding and inflammatory complications.

## 4. Conclusion

The use of an erbium laser is a modern and effective method for the dental surgical treatment of patients with primary immune thrombocytopenia. This technology makes possible to provide surgical dental care in this group of patients at a qualitatively new level in outpatient settings, without prolonged medical preparation.
